# Supplementary material for: Social isolation and cardiometabolic burden synergistically predict physical dysfunction in aging Chinese adults: Evidence of risk thresholds and the mediating role of frailty
Source: PLoS One. 2025 Oct 29;20(10):e0335467. doi: 10.1371/journal.pone.0335467 (PMC12571318; doi:10.1371/journal.pone.0335467)
Supplement: S1 Table — (DOCX) [file pone.0335467.s003.docx]

**Supplementary Table 1 Sensitivity analysis of threshold effect analysis**

| Exposure | Model/Segment | OR (95% CI) | E-value point estimation value | Lower bound of E-value confidence interval |
| --- | --- | --- | --- | --- |
| VAI | Model I: A straight line effect | 1.008 (0.989, 1.029) 0.405 | 1.07 | 1.02 |
|  | Model II: <K-segment (VAI < 13.053) | 1.032 (1.002, 1.063) 0.038 | 1.14 | 1.09 |
|  | Model II: ≥ K-segment (VAI ≥ 13.053 | 0.964 (0.920, 1.009) 0.112 | 1.16 | 1.14 |
| eGFR | Model I: A straight line effect | 0.995 (0.990, 1.001) 0.077 | 1.05 | 1.02 |
|  | Model II: <K-segment (AIP < 70.958) | 1.006 (0.993, 1.019) 0.384 | 1.06 | 1.02 |
|  | Model II: ≥ K-segment (AIP ≥ 70.958) | 0.990 (0.982, 0.998) 0.013 | 1.08 | 1.03 |
| Frailty Index | Model I: A straight line effect | 2.167 (2.037, 2.305) <0.001 | 2.31 | 1.77 |
|  | Model II: <K-segment (NHDL < 7.679) | 2.124 (1.993, 2.264) <0.001 | 2.27 | 1.75 |
|  | Model II: ≥ K-segment (NHDL ≥ 7.679) | NA |  |  |
| ASM | Model I: A straight line effect | 1.001 (0.971, 1.031) 0.967 | 1.02 | 1.00 |
|  | Model II: <K-segment (RC < 22.94) | 0.972 (0.938, 1.008) 0.124 | 1.13 | 1.10 |
|  | Model II: ≥ K-segment (RC ≥ 22.94) | 1.166 (1.041, 1.307) 0.008 | 1.37 | 1.30 |
| CTI | Model I: A straight line effect | 1.122 (1.024, 1.230) 0.013 | 1.31 | 1.27 |
|  | Model II: <K-segment (eGFR < 10.348) | 1.182 (1.066, 1.311) 0.002 | 1.40 | 1.30 |
|  | Model II: ≥ K-segment (eGFR ≥ 10.348) | 0.586 (0.319, 1.078) 0.086 | 1.94 | 1.57 |
| Estimated pulse wave velocity | Model I: A straight line effect | 1.243 (1.172, 1.319) <0.001 | 1.47 | 1.34 |
|  | Model II: <K-segment (FI < 7.178) | 2.026 (1.113, 3.690) 0.021 | 2.20 | 1.71 |
|  | Model II: ≥ K-segment (FI ≥ 7.178) | 1.226 (1.153, 1.304) <0.001 | 1.45 | 1.33 |
