# Supplementary material for: Social isolation and cardiometabolic burden synergistically predict physical dysfunction in aging Chinese adults: Evidence of risk thresholds and the mediating role of frailty
Source: PLoS One. 2025 Oct 29;20(10):e0335467. doi: 10.1371/journal.pone.0335467 (PMC12571318; doi:10.1371/journal.pone.0335467)
Supplement: Attachment — Submitted filename: Response to Reviewers.docx [file pone.0335467.s005.docx]

**Point-by-point Response Letter-[Manuscript ID:PONE-D-25-39258]**

Title:**Social Isolation and Cardiometabolic Burden Synergistically Predict Physical Dysfunction in Aging Chinese Adults: Evidence of Risk Thresholds and the Mediating Role of Frailty**

**Dear Editors and Reviewers of Plos One,**

We are deeply grateful for considering our manuscript for ***Plos One*** and value the insightful comments offered. These suggestions have been crucial for enhancing our manuscript's quality, and we're confident the revisions have significantly improved its clarity, rigor, and impact. We've meticulously revised the manuscript, addressing each comment with careful consideration and research. Besides responding to the feedback, we've also thoroughly reviewed the entire manuscript, making additional refinements to ensure top - notch scientific and literary quality. All changes are clearly tracked in the revised manuscript, and a clean version is also prepared. Below are our detailed point-by-point responses to each comment. We've aimed to be comprehensive and transparent in our explanations, providing evidence and reasoning for the changes made. Thank you again sincerely for your dedication and hard work. Your expertise is invaluable to the scientific community, and we're honored to contribute to this prestigious journal.

Yours Sincerely,

ZhongQiang Guo

Henan University

**[guozhongqiang0701@gmail.com](mailto:guozhongqiang0701@gmail.com)**

**Editor**

**Comments/suggestions:
1:Title**

**Response:**Thank you for the valuable feedback from the editor,The updated title highlights the core variables (Social Isolation, CKM, Physical Dysfunction, Frailty) and main findings (Mediating Role, Risk Thresholds)

**Revised version:** Social isolation, cardiometabolic syndrome, and physical dysfunction in aging chinese adults: the pivotal mediating role of frailty and evidence of risk thresholds

**2:keywords**

**Response:**Thank you for the valuable feedback from the editor,Deleted the typo "pulse circulation disorders" and replaced it with the overly broad "lipid metabolism disorders"

**Revised version:** Modified keywords:social isolation;cardiovascular-kidney-metabolic (CKM) syndrome;frailty;physical dysfunction;threshold effect;CHARLS.

**3: Abbreviations**

**Response:**We sincerely thank the editor for this important comment. We have thoroughly reviewed and standardized the use of abbreviations throughout the manuscript to ensure clarity and consistency for readers. The specific measures we have taken are as follows:

First Use Definition: Every abbreviation is now explicitly defined upon its first appearance in the main text, including the Abstract, Introduction, Methods, Results, and Discussion. The format used is "Full Term (Abbreviation)".

Example from the Abstract: "...cardiovascular–kidney–metabolic (CKM) syndrome... estimated pulse wave velocity [EPWV]..."

Creation of a Standalone Abbreviations Section: We have added a dedicated "Abbreviations" section in the manuscript, located after the "Acknowledgments" section and before the "References" list. This section provides an alphabetical list of all major abbreviations used in the paper for quick reference.

Consistency Check: We have ensured that each abbreviation is used consistently after its initial definition and that no undefined abbreviations appear in the text.

We believe these revisions have significantly improved the readability of the manuscript and prevented any potential confusion regarding the terminology used.

**Revised version:** PD, Physical Dysfunction; SI, Social Isolation; EPWV, Estimated Pulse Wave Velocity; ASM, Accessory Skeletal Muscle Mass; VAI, Visceral Adiposity Index; eGFR, Estimated Glomerular Filtration Rate; CTI, C-reactive protein triglyceride glucose index.

**4: Scoring, e.g., Likert 4**

**Response:**We thank the editor for raising this crucial point regarding the clarity of our scoring methods. We have comprehensively revised the manuscript to provide explicit and detailed descriptions of how all key composite variables were constructed and scored. The specific clarifications are as follows:

**Revised version:** **(lines 170-269) in the “Materials and methods” section.**

**Outcome variable**

**Physical dysfunction**

Calculation formula and assignment rules: The evaluation items include 9 basic physical function activities: running or jogging for 1 kilometre; walking 1 kilometre; walking 100 metres; rising after sitting for a long time; continuously climbing several layers of stairs; bending or squatting; raising arms over shoulders; picking up small coins from a table; and carrying heavy objects (>10 pounds). Answer options and coding: 0 for no difficulty, 1 for difficulty but still able to complete, 2 for difficulty and help required, and 3 for inability to complete. If a subject reported difficulty (coding ≥ 1) in any of the 9 items, this was defined as physical dysfunction [24].

**Core independent variables**

Social Isolation (SI): The construction in CHARLS research is based on five core items: marital status (1 point for unmarried, divorced, or widowed), living arrangement (1 point for living alone), social contact frequency with children (1 point for less than once a week), type of residence (1 point for rural areas), and participation in social activities (1 point for not participating in any social activities during the past month).

If each item met the conditions, it was given a score of 1 point. The total score ranges from 0 to 5 points. The higher the score is, the more serious the social isolation.

Finally, a total score of ≥ 2 points is considered as the critical point for judging social isolation (i.e., "yes"), and a total score of <2 points is judged as non-social isolation ("no").

This scoring standard refers to the international health and retirement research (HRS) model, and each item is simply summed with equal weight (i.e., 1 point for each item) [25].

This critical value (≥ 2 points) has been verified in many international studies to effectively predict health outcomes such as mortality, cardiovascular disease, and chronic kidney disease. For example, a meta-analysis [26,27] revealed that social isolation increased the risk of all-cause death by 29% (OR=1.29), coronary heart disease by 29%, and stroke by 32%.

In the Chinese population, the CHARLS cohort study (the sample covers middle-aged and elderly people in 28 provinces across the country) further confirmed [25] that the risk of renal function decline in social isolation with the same critical value (≥ 2 points) significantly increased, indicating that the standard has predictive validity for the Chinese population.

With respect to the processing of missing data, CHARLS routinely uses statistical techniques to fill in missing values (such as mode for classified variables and mean for continuous variables) and corrects sampling deviation by analysing weights to ensure national representativeness [28,29].

However, in the specific operation of the social isolation component, if the data of five core items are missing, this study chooses to directly eliminate the sample rather than to complete it.

This approach may reduce the sample size but avoid the classification bias that may be introduced by imputation, such as the lack of marital status or social activity participation. If the mode is filled, some actual isolators may be erroneously classified into a nonisolated group [25].

Of note [30], mentioned that other studies used different threshold values (such as ≥ 4 points to determine isolation), but CHARLS mainly used the international standard of ≥ 2 points and noted that the reliability and validity of this standard in the elderly population has been supported by many studies [31].

**Cardiovascular–kidney–metabolic (CKM) syndrome stages**

Stage 0: no CKM risk factors (no overweight/obesity, metabolic abnormalities, CKD, or cardiovascular disease).

Stage 1: overweight/obesity (BMI ≥ 25 kg/m²) or abdominal obesity but no other metabolic risk factors (such as hypertension or diabetes) or CKD.

Stage 2: presence of metabolic risk factors (hypertension, hypertriglyceridaemia, metabolic syndrome, diabetes) or CKD (eGFR<60 mL/min/1.73 m² or proteinuria) but no cardiovascular disease.

Stage 3: at least one case of subclinical cardiovascular disease (such as atherosclerosis or subclinical heart failure) accompanied by overweight/obesity, metabolic risk factors, or CKD.

Stage 4: clinically diagnosed cardiovascular diseases (such as coronary heart disease, heart failure, and stroke) with at least one other risk factor (CKD, BMI ≥ 25 kg/m², diabetes, hypertension, etc.) are divided into no renal failure (4a) and renal failure (4b, eGFR<15 mL/min/1.73 m² or renal replacement therapy) [32,33].

**Vascular ageing biomarkers**

**Estimated pulse wave velocity (EPWV)**

EPWV is calculated on the basis of the MBP and age using the following formula: EPWV$\text{=9.587-(0.402×}$age$\text{)+(4.560×}\text{10}^{\text{-3}}\text{×}$age$\text{ }^{\text{2}}\text{)-(2.621×}\text{10}^{\text{-5}}\text{×}$age$\text{ }^{\text{2}}\text{×}$MBP) $\text{+(3.176×}\text{10}^{\text{-3}}\text{×}$ age $\text{×}$ MBP$\text{)-(1.832×}\text{10}^{\text{-2}}\text{×}$ MBP), where $\text{MBP=DBP+0.4}\left( \text{SBP-DBP} \right)$ [34].

Body Composition Indicators:

Accessory skeletal muscle mass (ASM): $\text{ASM=0.193×weight（kg）+0.107×height（cm）-4.157×gender-0.037×age-2.631}$Male sex=1, female sex=2, sum of skeletal muscle mass in arms and legs (kg), and assessment of muscle mass [35].

Frailty Index=$\frac{\text{Number of health deficits present}}{\text{Total number of deficits assessed}}$, which is a comprehensive assessment of individual physiological reserve decline and vulnerability [36].

Frailty: Using a Fried phenotypic model, weakness was defined as a score ≥ 3 for the following five criteria: involuntary weight loss (weight loss ≥ 4.5 kg or 5% in the past year), self-reported fatigue (CES-D assessment), a decrease in grip strength (BMI and sex correction), slow walking speed (4-metre walking test), and low physical activity (IPAQ questionnaire assessment) [37].

When scoring, each criterion is a binary variable (existence=1 point; nonexistence=0 points). If the total score is ≥ 3 points, it is judged as frailty, and if the total score is <3 points, it is judged as non-frailty.

(yes/no) [38].

Visceral Adiposity Index (VAI):

$$\text{VAI}_{\text{men}}\text{=}\left( \frac{\text{Waist circumference}}{\text{39.68+}\left( \text{1.88×BMI} \right)} \right)\text{×}\left( \frac{\text{Triglycerides}}{\text{1.03}} \right)\text{×}\left( \frac{\text{1.31}}{\text{HDL-C}} \right)$$

$$\text{VAI}_{\text{women}}\text{=}\left( \frac{\text{Waist circumference}}{\text{36.58+}\left( \text{1.89×BMI} \right)} \right)\text{×}\left( \frac{\text{Triglycerides}}{\text{0.81}} \right)\text{×}\left( \frac{\text{1.52}}{\text{HDL-C}} \right)$$

Indicators reflecting visceral fat function and quantity were calculated on the basis of waist circumference, body mass index, triglyceride levels, and high-density lipoprotein cholesterol levels [39].

Core indicators of renal function

Estimated Glomerular Filtration Rate, eGFR:

Female:

$$\text{• Scr≤62 μmol/L→eGFR=144×}\left( \text{Scr/62} \right)^{\text{-0}}\text{⋅}^{\text{329}}\text{×0.993Age}$$

$$\text{• Scr>62 μmol/L→eGFR=144×}\left( \text{Scr/62} \right)^{\text{-1}}\text{⋅}^{\text{209}}\text{×0.993Age}$$

Male:

$$\text{• Scr≤80 μmol/L→eGFR=141×}\left( \text{Scr/80} \right)^{\text{-0}}\text{⋅}^{\text{411}}\text{×0.993Age}$$

$$\text{• Scr>80 μmol/L→eGFR=141×}\left( \text{Scr/80} \right)^{\text{-1}}\text{⋅}^{\text{209}}\text{×0.993Age}$$

followed by the assessment of renal function [40].

**Lipid metabolism indicators**

C-reactive protein triglyceride glucose index (CTI): The formula for detecting C-reactive protein, triglyceride, and glucose levels in blood samples is as follows:

$\text{CTI=0.412 × ln(C-reactive protein [CRP]) + ln(triglycerides [mg/dL] × fasting blood glucose [mg/dL]/2) [41]}$. As a novel composite biomarker that integrates inflammation and lipid metabolism, CTI has shown significant potential in predicting neurodegenerative diseases [42].

**References**

24.	Resnik L, Gozalo P, Hart DL. Weighted index explained more variance in physical function than an additively scored functional comorbidity scale. J Clin Epidemiol. 2011;64: 320-330.

25.	Zhou W, Li Y, Ning Y, Gong S, Song N, Zhu B, et al. Social isolation is associated with rapid kidney function decline and the development of chronic kidney diseases in middle-aged and elderly adults: findings from the China health and retirement longitudinal study (CHARLS). Front Med (Lausanne). 2021;8: 782624.

26.	Valtorta NK, Kanaan M, Gilbody S, Ronzi S, Hanratty B. Loneliness and social isolation as risk factors for coronary heart disease and stroke: systematic review and meta-analysis of longitudinal observational studies. Heart. 2016;102: 1009-1016.

27.	Holt-Lunstad J, Smith TB, Baker M, Harris T, Stephenson D. Loneliness and social isolation as risk factors for mortality: a meta-analytic review. Perspect Psychol Sci. 2015;10: 227-237.

28.	Zhao Y, Hu Y, Smith JP, Strauss J, Yang G. Cohort profile: the China health and retirement longitudinal study (CHARLS). Int J Epidemiol. 2014;43: 61-68.

29.	Song Y, Zhu C, Shi B, Song C, Cui K, Chang Z, et al. Social isolation, loneliness, and incident type 2 diabetes mellitus: results from two large prospective cohorts in Europe and East Asia and Mendelian randomization. EClinicalMedicine. 2023;64: 102236.

30.	Qin W, Xiang X, Taylor H. Driving cessation and social isolation in older adults. J Aging Health. 2020;32: 962-971.

31.	Lyu C, Siu K, Xu I, Osman I, Zhong J. Social isolation changes and long-term outcomes among older adults. JAMA Netw Open. 2024;7: e2424519.

32.	Roa BPA, Varne SRR. Evaluation of cardiovascular kidney metabolic syndrome and cardiovascular hepato-renal metabolic syndrome (CHARM): from encryption to decryption. Int J Clin Exp Physiol. 2024;10: 98-102.

33.	Theodorakis N, Nikolaou M. From cardiovascular-kidney-metabolic syndrome to cardiovascular-renal-hepatic-metabolic syndrome: proposing an expanded framework. Biomolecules. 2025;15: 213.

34.	Li D, Cao F, Cheng W, Xu Y, Yang C. Predictive value of estimated pulse wave velocity for cardiovascular and all-cause mortality in individuals with obesity. Diabetol Metab Syndr. 2023;15: 40.

35.	Ji W, Liu X, Zhang Y, Zhao Y, He Y, Cui J, et al. Development of formulas for calculating L3 skeletal muscle mass index and visceral fat area based on anthropometric parameters. Front Nutr. 2022;9: 910771.

36.	Rockwood K, Nassar B, Mitnitski A. Apolipoprotein E-polymorphism, frailty and mortality in older adults. J Cell Mol Med. 2008;12: 2754-2761.

37.	Bautista MAC, Malhotra R. Identification and measurement of frailty: a scoping review of published research from Singapore. Ann Acad Med Singap. 2018;47: 455-491.

38.	Quach J, Kehler DS, Giacomantonio N, McArthur C, Blanchard C, Firth W, et al. Association of admission frailty and frailty changes during cardiac rehabilitation with 5-year outcomes. Eur J Prev Cardiol. 2023;30: 807-819.

39.	Kaminska MS, Lubkowska A, Panczyk M, Walaszek I, Grochans S, Grochans E, et al. Relationships of body mass index, relative fat mass index, and waist circumference with serum concentrations of parameters of chronic inflammation. Nutrients. 2023;15: 2789.

40.	Yu S, Yang H, Guo X, Zheng L, Sun Y. Association between obese phenotype and mildly reduced eGFR among the general population from rural Northeast China. Int J Environ Res Public Health. 2016;13: 540.

41.	Ren Y, Xu R, Zhang J, Jin Y, Zhang D, Wang Y, et al. Association between the C-reactive protein-triglyceride-glucose index and endometriosis: a cross-sectional study using data from the national health and nutrition examination survey, 1996-2006. BMC Womens Health. 2025;25: 13.

42.	Li Q, Song Y, Zhang Z, Xu J, Liu Z, Tang X, et al. The combined effect of triglyceride-glucose index and high-sensitivity C-reactive protein on cardiovascular outcomes in patients with chronic coronary syndrome: a multicenter cohort study. J Diabetes. 2024;16: e13589.

**5: phases of CKM**

**Response:**

We thank the editor for this critical comment. We acknowledge that the original manuscript lacked a detailed definition of the cardiovascular-kidney-metabolic (CKM) syndrome staging, which is essential for understanding the exposure gradient. In response, we have now provided a comprehensive and clear description of the CKM staging criteria in the revised manuscript.

Specifically, in the "Variable Definition and Measurement" section (Section 2.2.2, under "Core independent variables"), we have added a new subsection titled "Cardiovascular–kidney–metabolic (CKM) syndrome stages". This subsection details the operational definitions for all five stages (0 to 4), based on the seminal framework proposed by the American Heart Association (AHA) and subsequent operationalizations for epidemiological studies [32, 33].

**Revised version:** **(lines 217-230) in the “Materials and methods” section.**

Cardiovascular–kidney–metabolic (CKM) syndrome stages

Stage 0: no CKM risk factors (no overweight/obesity, metabolic abnormalities, CKD, or cardiovascular disease).

Stage 1: overweight/obesity (BMI ≥ 25 kg/m²) or abdominal obesity but no other metabolic risk factors (such as hypertension or diabetes) or CKD.

Stage 2: presence of metabolic risk factors (hypertension, hypertriglyceridaemia, metabolic syndrome, diabetes) or CKD (eGFR<60 mL/min/1.73 m² or proteinuria) but no cardiovascular disease.

Stage 3: at least one case of subclinical cardiovascular disease (such as atherosclerosis or subclinical heart failure) accompanied by overweight/obesity, metabolic risk factors, or CKD.

Stage 4: clinically diagnosed cardiovascular diseases (such as coronary heart disease, heart failure, and stroke) with at least one other risk factor (CKD, BMI ≥ 25 kg/m², diabetes, hypertension, etc.) are divided into no renal failure (4a) and renal failure (4b, eGFR<15 mL/min/1.73 m² or renal replacement therapy) [32,33].

**References**

32.	Roa BPA, Varne SRR. Evaluation of cardiovascular kidney metabolic syndrome and cardiovascular hepato-renal metabolic syndrome (CHARM): from encryption to decryption. Int J Clin Exp Physiol. 2024;10: 98-102.

33.	Theodorakis N, Nikolaou M. From cardiovascular-kidney-metabolic syndrome to cardiovascular-renal-hepatic-metabolic syndrome: proposing an expanded framework. Biomolecules. 2025;15: 213.

**6: figures and Tables are not clear and tables missed footnotes**

**Response:**We sincerely thank the editor for this vital feedback regarding the presentation of our data. We have thoroughly revised all figures and tables to enhance their clarity, professionalism, and information value. The specific actions taken are detailed below:

**Revised version:**

**Table 1. Baseline Characteristics of the Population Stratified by PD (n=3756).**

**Footnote:**The data is expressed as mean ± standard deviation or frequency (percentage).

**Table 2. Multiple Logistic Regression Equation.**

**Footnote:**Data in the table: β (95%CI) Pvalue / OR (95%CI) Pvalue 
Result variable:Physical dysfunction 
Exposed variables:Social Isolation;VAI;eGFR;Frailty Index;Frailty;ASM;CTI;CKM;Estimated pulse wave velocity 
Non-adjusted model adjust for: None 
Adjust I model adjust for: AGE; Gender
Adjust II model adjust for:Living Area;Married Status;Education;Smoking status; Drinking Status

**Table 3. Threshold Effect Analysis.**

**Footnote:**Data in the table: β (95%CI) Pvalue / OR (95%CI) Pvalue

Result variable:Physical dysfunction

Exposed variables:VAI;eGFR;Frailty Index;ASM;CTI;Estimated pulse wave velocity

Adjust variables:AGE;Gender;Living Area;Married Status;Education;Smoking status;Drinking Status

**Fig 1. Flow Diagram for Participant Inclusion in the Study.**

**Fig 2. Smooth Curve Fitting.**

**Footnote:**Smooth curve fitting relationships were determined between (A) the VAI and physical dysfunction, (B) the eGFR and physical dysfunction, (C) the frailty index and physical dysfunction, (D) the ASM and physical dysfunction, (E) the CTI and physical dysfunction, and (F) the estimated pulse wave velocity and physical dysfunction.

**Fig 3. Subgroup Analysis.**

**Fig 4. Causal Mediation Analysis.**

**Footnote:**(A) Mediating effect analysis of social isolation as an indicator of estimated pulse wave velocity as it relates to physical dysfunction. (B) Mediating effect analysis of the frailty index as an indicator of estimated pulse wave velocity as it relates to physical dysfunction. (C) Mediating effect analysis of estimated pulse wave velocity as an indicator of the frailty index as it relates to physical dysfunction. (D) Mediating effect analysis of the estimated pulse wave velocity as an indicator of the CKM as it relates to physical dysfunction.

**7: statistical analysis: rephrase to be clear**

**Response:**

We are grateful to the editor for this essential comment. We fully agree that a clearly articulated statistical approach is fundamental. In response, we have completely rewritten and reorganized the "Statistical Analysis" section to enhance its clarity, logical flow, and methodological rigor. The key improvements are summarized as follows:

**Revised version:** **(lines 279-316) in the “Statistical analysis” section.**

**Statistical analysis**

Descriptive statistics were used to summarize the demographic, behavioural, and clinical characteristics and biomarker profiles of the study participants. Continuous variables with a normal distribution are presented as the mean ± standard deviation (SD), whereas categorical variables are expressed as frequencies and percentages (%). To compare baseline characteristics between groups with and without physical dysfunction, Pearson’s chi-square tests were employed for categorical variables, and independent Student’s t tests or one-way analysis of variance (ANOVA) were used for continuous variables, as appropriate [50,51].

All the statistical analyses were performed using R software (version 4.2.2). A two-sided p value < 0.05 was considered to indicate statistical significance for all tests.

The associations between core exposures (social isolation and multidimensional biomarkers, including VAI, eGFR, frailty index, ASM, CKM syndrome stage, and EPWV) and physical dysfunction (binary outcome) were assessed using multivariable logistic regression. The results are reported as odds ratios (ORs) with 95% confidence intervals (CIs). Two adjusted models were constructed: Model I was adjusted for basic demographic factors (age and sex), and Model II was further adjusted for lifestyle factors (smoking status and alcohol consumption) and socioeconomic factors (education level, marital status, and residential area) [52,53].

Stratification analyses were conducted to examine potential effect modifications for key subgroups, including age (<60 vs. ≥60 years), sex, residential area, marital status, education level, smoking history, and drinking frequency. Interaction terms were introduced into the logistic models, and subgroup-specific ORs were reported [54].

To explore the potential nonlinear relationships between continuous biomarkers (VAI, eGFR, frailty index, ASM, CTI, and EPWV) and physical dysfunction, a preliminary analysis was conducted by smooth curve fitting after adjusting for covariates [55]. If the visual inspection indicates that there is a nonlinear correlation, the two piecewise linear regression models [56] are applied to identify the inflection points through the iterative method system. The determination of the optimal threshold is based on the principle of maximizing the goodness of fit of the model, and the log likelihood ratio test is used to verify the statistical significance of the threshold effect. This method does not rely on a treatment group/control group design and is suitable for observational data analysis [57]. Its goal is to realize the objective determination and verification of critical biomarker values through the combination of nonlinear modelling and statistical tests.

Causal mediation analysis was carried out using the R package “mediation” with 1000 bootstrap simulations to estimate the direct, indirect, and total effects of key pathways—for instance, the mediating role of the frailty index in the association between EPWV and physical dysfunction. All the mediation models were adjusted for the full set of covariates (age, sex, residence, marital status, education, smoking status, and drinking status). The mediation proportion was calculated as the ratio of the indirect effect to the total effect. Bias-corrected bootstrap confidence intervals were reported for the mediation effects [50,51].

**References**

51.	Lee CS, Bowman M, Wu JL. Preliminary outcomes from a single-session, asynchronous, online stress and anxiety management workshop for college students. Trends Psychiatry Psychother. 2023;45: e20210448.

52.	Polyvianaia M, Stepanova N, Yachnik Y, Myshakivska O, Fegert JM, Sitarski E, et al. Mental health support needs of students during the wartime. Proceeding Shevchenko Sci Soc Med Sci. 2024;76: 1-17.

53.	Policarpo S, Carvalhana S, Craciun A, Crespo RR, Cortez-Pinto H. Do MAFLD patients with harmful alcohol consumption have a different dietary intake? Nutrients. 2022;14: 1335.

54.	Guo M, Xu S, He X, He J, Yang H, Zhang L. Decoding emotional resilience in aging: unveiling the interplay between daily functioning and emotional health. Front Public Health. 2024;12: 1391033.

55.	Li Z, Huang Y, Patil D, Sanda MG. Covariate adjustment in continuous biomarker assessment. Biometrics. 2023;79: 39-48.

56.	Boileau P, Qi NT, van der Laan MJ, Dudoit S, Leng N. A flexible approach for predictive biomarker discovery. Biostatistics. 2023;24: 1085-1105.

57.	van der Laan MJ. Targeted maximum likelihood based causal inference: part I. Int J Biostat. 2010;6: 2.

**8: discussion is weak**

**Response:**We sincerely thank the editor for this critical comment, which has helped us significantly strengthen the interpretation and contextualization of our findings. In response, we have undertaken a substantial and comprehensive revision of the "Discussion" section to move beyond a mere restatement of results and towards a more critical, integrative, and impactful scientific narrative. The key enhancements include:

Strengthened Contextualization with Global Literature: We have integrated recent and high-quality evidence from global cohorts to frame our findings, moving beyond a purely Chinese context. For instance, we now cite studies by Mehrabi & Béland (2021) on social isolation and frailty, and Fried et al. (2001) on the frailty phenotype, to position our work within the international scientific discourse and demonstrate the broader relevance of our results.

Deeper Interpretation of Key Findings, Particularly the Mediation Result:

We explicitly acknowledge and discuss the potential for overestimation of the large mediation effect (57.8%) due to possible conceptual overlap between the frailty index and the physical dysfunction outcome, framing this as a consideration for interpretation rather than ignoring it.

We contrast our finding with other mediation studies (e.g., Bai et al., 2024, who found a lower mediation for depressive symptoms) to highlight the unique and central role of frailty in the pathway from vascular aging to physical disability in our study.

Explicit Acknowledgment of Limitations and Future Directions: We have expanded the limitations paragraph into a robust and critical self-assessment. This now includes:

A clear discussion of the cross-sectional design's fundamental limitation for causal inference.

The introduction of the concept of bidirectionality between vascular ageing and frailty.

A consideration of how societal changes since the 2015 data collection might affect generalizability.

An acknowledgment of potential residual confounding by unmeasured factors like detailed physical activity and nutrition.

In-depth Exploration of Subgroup Findings: We moved beyond simply reporting subgroup differences (e.g., stronger effects in males <60 years) to proposing plausible sociocultural and economic explanations (e.g., gender roles, occupational patterns, migration history) for these heterogeneities. This adds a rich, mechanistic layer to the discussion.

Emphasis on Clinical and Public Health Implications: We have more forcefully articulated the novelty and clinical utility of the identified biomarker thresholds (e.g., EPWV = 7.178 m/s), framing them as actionable intervention windows. We also elaborate on the concept of "social prescription" and integrated strategies that combine social and biological interventions.

Improved Logical Flow and Narrative: The entire section has been restructured to tell a more compelling story: from confirming independent effects, to unveiling synergistic risks and key thresholds, to elucidating the central mediating pathway, and finally to discussing limitations and translating these insights into meaningful implications for research and practice.

We believe the revised Discussion now provides a critical, balanced, and insightful synthesis of our findings, firmly establishing their significance within the field and outlining a clear path for future research.

**Reviewer 1**

**Comments/suggestions:**

**1. Study Design and Causality
- The manuscript acknowledges the cross-sectional nature of the study but still makes causal inferences (e.g., “EPWV → frailty → physical dysfunction” as a pathway). The language in the results and discussion should be moderated to avoid implying causality without longitudinal validation.
- Consider clarifying that mediation analyses in cross-sectional data can only suggest potential pathways and require prospective confirmation.**

**Response:**

We sincerely thank the reviewer for this crucial comment regarding the interpretation of our findings. We fully agree that the cross-sectional nature of our data necessitates caution in inferring causality. In response, we have meticulously revised the manuscript to ensure that our language is appropriate and does not overstate the causal implications of our results.

Furthermore, to ensure the highest standard of academic English and to guarantee that our intended meaning is conveyed with precision and clarity, the entire manuscript has undergone professional language editing by American Journal Experts (AJE). This process has helped us to carefully refine the wording throughout the text.

The specific actions taken to address this comment are as follows:

Systematic Moderation of Causal Language: We have systematically reviewed and moderated the language throughout the manuscript, particularly in the Results and Discussion sections. We have replaced causal terms like "effect", "impact", and "lead to" with more tentative and associative language such as "association", "relationship", "link", and "pathway". For instance, the pathway is now described as the "association between EPWV and physical dysfunction mediated by frailty" rather than a direct causal chain.

Explicit Clarification on Mediation Analysis: In the Statistical Analysis section (2.3), we have added an explicit statement to clarify the interpretative limits of our mediation analysis. The text now clearly states:

"It is important to note that mediation analyses conducted on cross-sectional data can only suggest potential causal pathways and mechanistic hypotheses; they cannot confirm causality or establish temporal precedence, which requires prospective longitudinal validation."

Strengthened Discussion on Limitations: We have expanded the Discussion section to explicitly and emphatically acknowledge this limitation. We now state that the identified pathways (e.g., EPWV → frailty → physical dysfunction) represent plausible biological mechanisms that are consistent with existing longitudinal literature, but that they must be interpreted as hypotheses generated by our study, not as proven causal relationships. We have explicitly called for future longitudinal studies to confirm the temporal sequence and causal nature of these associations.

By implementing these changes and leveraging professional editing, we believe we have adequately addressed the reviewer's concern, presenting our findings with the necessary scientific caution while still highlighting their potential significance for generating future hypotheses.

**2. Operationalization of Social Isolation
- The social isolation score derivation requires more detail. Please specify which CHARLS items were used, their weighting, and whether the measure has been validated in the Chinese population.
- Indicate how missing data in social isolation components were handled, as imputation could affect classification.**

**Response:**

We thank the reviewer for this important request for clarification. We have comprehensively revised the manuscript to provide a detailed and transparent account of the construction and validation of the social isolation score, as well as the handling of missing data.

**Revised version:** **(lines 182-215) in the “Variable definition and measurement” section.**

Core independent variables

Social Isolation (SI): The construction in CHARLS research is based on five core items: marital status (1 point for unmarried, divorced, or widowed), living arrangement (1 point for living alone), social contact frequency with children (1 point for less than once a week), type of residence (1 point for rural areas), and participation in social activities (1 point for not participating in any social activities during the past month).

If each item met the conditions, it was given a score of 1 point. The total score ranges from 0 to 5 points. The higher the score is, the more serious the social isolation.

Finally, a total score of ≥ 2 points is considered as the critical point for judging social isolation (i.e., "yes"), and a total score of <2 points is judged as non-social isolation ("no").

This scoring standard refers to the international health and retirement research (HRS) model, and each item is simply summed with equal weight (i.e., 1 point for each item) [25].

This critical value (≥ 2 points) has been verified in many international studies to effectively predict health outcomes such as mortality, cardiovascular disease, and chronic kidney disease. For example, a meta-analysis [26,27] revealed that social isolation increased the risk of all-cause death by 29% (OR=1.29), coronary heart disease by 29%, and stroke by 32%.

In the Chinese population, the CHARLS cohort study (the sample covers middle-aged and elderly people in 28 provinces across the country) further confirmed [25] that the risk of renal function decline in social isolation with the same critical value (≥ 2 points) significantly increased, indicating that the standard has predictive validity for the Chinese population.

With respect to the processing of missing data, CHARLS routinely uses statistical techniques to fill in missing values (such as mode for classified variables and mean for continuous variables) and corrects sampling deviation by analysing weights to ensure national representativeness [28,29].

However, in the specific operation of the social isolation component, if the data of five core items are missing, this study chooses to directly eliminate the sample rather than to complete it.

This approach may reduce the sample size but avoid the classification bias that may be introduced by imputation, such as the lack of marital status or social activity participation. If the mode is filled, some actual isolators may be erroneously classified into a nonisolated group [25].

Of note [30], mentioned that other studies used different threshold values (such as ≥ 4 points to determine isolation), but CHARLS mainly used the international standard of ≥ 2 points and noted that the reliability and validity of this standard in the elderly population has been supported by many studies [31].

**References**

25.	Zhou W, Li Y, Ning Y, Gong S, Song N, Zhu B, et al. Social isolation is associated with rapid kidney function decline and the development of chronic kidney diseases in middle-aged and elderly adults: findings from the China health and retirement longitudinal study (CHARLS). Front Med (Lausanne). 2021;8: 782624.

26.	Valtorta NK, Kanaan M, Gilbody S, Ronzi S, Hanratty B. Loneliness and social isolation as risk factors for coronary heart disease and stroke: systematic review and meta-analysis of longitudinal observational studies. Heart. 2016;102: 1009-1016.

27.	Holt-Lunstad J, Smith TB, Baker M, Harris T, Stephenson D. Loneliness and social isolation as risk factors for mortality: a meta-analytic review. Perspect Psychol Sci. 2015;10: 227-237.

28.	Zhao Y, Hu Y, Smith JP, Strauss J, Yang G. Cohort profile: the China health and retirement longitudinal study (CHARLS). Int J Epidemiol. 2014;43: 61-68.

29.	Song Y, Zhu C, Shi B, Song C, Cui K, Chang Z, et al. Social isolation, loneliness, and incident type 2 diabetes mellitus: results from two large prospective cohorts in Europe and East Asia and Mendelian randomization. EClinicalMedicine. 2023;64: 102236.

30.	Qin W, Xiang X, Taylor H. Driving cessation and social isolation in older adults. J Aging Health. 2020;32: 962-971.

31.	Lyu C, Siu K, Xu I, Osman I, Zhong J. Social isolation changes and long-term outcomes among older adults. JAMA Netw Open. 2024;7: e2424519.

**3.Biomarker Threshold Determination**

**- The approach for identifying inflection points (threshold effect analysis) should be described more explicitly. Were spline regressions, piecewise linear models, or other non-linear models used? How was the optimal cut-point determined and validated?**

**- Consider presenting sensitivity analyses to show robustness of thresholds.**

**Response:**

We sincerely thank the reviewer for these insightful questions, which have allowed us to significantly strengthen the methodological transparency and robustness of our threshold effect analysis. We have thoroughly revised the "Statistical Analysis" section to provide a more explicit, step-by-step description of our approach. Furthermore, we have conducted additional sensitivity analyses, including the calculation of E-values, to quantitatively assess the robustness of the identified thresholds.

The specific modifications and enhancements are as follows:

Explicit Description of the Analytical Approach:

We now explicitly state that we employed a two-piecewise linear regression model to objectively identify inflection points, distinguishing it from other non-linear approaches.

The revision clarifies that the optimal inflection point was determined through an iterative procedure that maximizes the model likelihood, with statistical significance formally tested using the log-likelihood ratio test against a simple linear model.

Presentation of Sensitivity Analyses for Robustness:

In direct response to the reviewer's suggestion to demonstrate robustness, we have conducted and now report E-value analyses.

As detailed in the new "Sensitivity analysis for threshold effects" subsection within the Methods, the E-value quantifies the minimum strength of unmeasured confounding required to explain away the observed association. The considerably large E-values calculated for our key results (e.g., 2.20 for EPWV and 2.27 for the Frailty Index) increase our confidence that the identified thresholds are robust.

We have integrated the interpretation of these E-values into the Discussion section, contextualizing their meaning relative to common confounders in epidemiological studies.

We believe that the detailed methodological explanation, combined with the quantitative robustness checks provided by the E-value analysis, provides a comprehensive and rigorous foundation for our threshold effect findings. These additions have been integrated throughout the revised manuscript and supplementary materials.

**Revised version:** **(lines 317-327) in the “Statistical analysis” section.**

**Sensitivity analysis for threshold effects**

To assess the robustness of the identified threshold associations to potential unmeasured confounding, we calculated E-values for the significant segments of the piecewise linear models (58). The E-value quantifies the minimum strength of association that an unmeasured confounder would need to have with both the exposure and the outcome, conditional on the measured covariates, to fully explain away the observed exposure-outcome association (59). We report both the point estimate of the E-value for the observed effect size and the E-value for the lower limit of the 95% confidence interval. Larger E-values indicate that stronger unmeasured confounding would be required to nullify the association, thus implying greater robustness of the finding. The detailed results of the E-value sensitivity analysis for all biomarkers are presented in Supplementary Table 1.

**References**

58.	VanderWeele TJ, Ding P. Sensitivity analysis in observational research: introducing the E-value. Ann Intern Med. 2017;167(4):268-74.

59.	Mathur MB, Ding P, Riddell CA, VanderWeele TJ. Web site and R package for computing E-values. Epidemiology. 2018;29(5):e45-7.

**Supplementary Table 1 Sensitivity analysis of threshold effect analysis**

| Exposure | Model/Segment | OR (95% CI) | E-value point estimation value | Lower bound of E-value confidence interval |
| --- | --- | --- | --- | --- |
| VAI | Model I: A straight line effect | 1.008 (0.989, 1.029) 0.405 | 1.07 | 1.02 |
|  | Model II: <K-segment (VAI < 13.053) | 1.032 (1.002, 1.063) 0.038 | 1.14 | 1.09 |
|  | Model II: ≥ K-segment (VAI ≥ 13.053 | 0.964 (0.920, 1.009) 0.112 | 1.16 | 1.14 |
| eGFR | Model I: A straight line effect | 0.995 (0.990, 1.001) 0.077 | 1.05 | 1.02 |
|  | Model II: <K-segment (AIP < 70.958) | 1.006 (0.993, 1.019) 0.384 | 1.06 | 1.02 |
|  | Model II: ≥ K-segment (AIP ≥ 70.958) | 0.990 (0.982, 0.998) 0.013 | 1.08 | 1.03 |
| Frailty Index | Model I: A straight line effect | 2.167 (2.037, 2.305) <0.001 | 2.31 | 1.77 |
|  | Model II: <K-segment (NHDL < 7.679) | 2.124 (1.993, 2.264) <0.001 | 2.27 | 1.75 |
|  | Model II: ≥ K-segment (NHDL ≥ 7.679) | NA |  |  |
| ASM | Model I: A straight line effect | 1.001 (0.971, 1.031) 0.967 | 1.02 | 1.00 |
|  | Model II: <K-segment (RC < 22.94) | 0.972 (0.938, 1.008) 0.124 | 1.13 | 1.10 |
|  | Model II: ≥ K-segment (RC ≥ 22.94) | 1.166 (1.041, 1.307) 0.008 | 1.37 | 1.30 |
| CTI | Model I: A straight line effect | 1.122 (1.024, 1.230) 0.013 | 1.31 | 1.27 |
|  | Model II: <K-segment (eGFR < 10.348) | 1.182 (1.066, 1.311) 0.002 | 1.40 | 1.30 |
|  | Model II: ≥ K-segment (eGFR ≥ 10.348) | 0.586 (0.319, 1.078) 0.086 | 1.94 | 1.57 |
| Estimated pulse wave velocity | Model I: A straight line effect | 1.243 (1.172, 1.319) <0.001 | 1.47 | 1.34 |
|  | Model II: <K-segment (FI < 7.178) | 2.026 (1.113, 3.690) 0.021 | 2.20 | 1.71 |
|  | Model II: ≥ K-segment (FI ≥ 7.178) | 1.226 (1.153, 1.304) <0.001 | 1.45 | 1.33 |

**Revised version:** **(lines 546-559) in the “Discussion” section.**

A key strength of our analysis is the evaluation of the robustness of the identified biomarker thresholds to potential unmeasured confounding. We employed E-values to quantify the strength of association an unmeasured confounder would need to have with both the biomarker and physical dysfunction to explain away the observed relationships(58). For the significant threshold associations—such as the effect of EPWV below 7.178 m/s (OR=2.026, E-value=2.20) and the frailty index below 7.679 (OR=2.124, E-value=2.27)—the E-values were substantively greater than 1. This indicates that to fully nullify these associations, an unmeasured confounder would need to be associated with both the exposure and the outcome by risk ratios of 2.20-fold and 2.27-fold, respectively, which is a relatively strong magnitude for common confounders. Similarly, the E-values for other biomarkers like ASM (E-value=1.37) and CTI (E-value=1.40) also suggest a degree of robustness, as they exceed the strengths of many measured confounders in epidemiological studies. While this does not preclude the possibility of residual confounding, it increases confidence that the non-linear relationships we identified are not easily attributable to a single, strong, unmeasured confounder.

**References**

58.	VanderWeele TJ, Ding P. Sensitivity analysis in observational research: introducing the E-value. Ann Intern Med. 2017;167(4):268-74.

**4.Confounding and Effect Modification
- While demographic, lifestyle, and socioeconomic covariates were adjusted for, residual confounding is likely. Variables such as depressive symptoms, physical activity, and nutritional status may influence both social isolation and physical function.
- Some effect modification findings (e.g., stronger effects in males <60 years) warrant deeper exploration — could occupational patterns, migration history, or gender norms explain this?**

**Response:**

We sincerely thank the reviewer for these insightful observations regarding residual confounding and effect modification. We have substantially revised our manuscript to address these important considerations with greater depth and rigor.

1. Addressing Potential Residual Confounding

We fully acknowledge the reviewer's concern about potential residual confounding from factors such as depressive symptoms, physical activity patterns, and nutritional status. While our analysis adjusted for a comprehensive set of demographic, lifestyle, and socioeconomic covariates, we agree that these additional factors could theoretically influence both social isolation and physical function.

To address this limitation more thoroughly, we have:

Added explicit acknowledgment of these potential confounding factors in the Discussion section, specifically noting that while variables like depressive symptoms were partially captured in our frailty assessment, more detailed measurements of physical activity intensity and nutritional quality were not fully available.

Implemented and reported E-value analyses to quantitatively assess the robustness of our key findings to potential unmeasured confounding. The substantial E-values for our primary associations (ranging from 1.37 to 2.27 for significant threshold effects) indicate that it would require strong, unmeasured confounding to fully explain away these relationships.

Emphasized the need for future studies to incorporate more detailed measurements of these potential confounders to further validate our findings.

2. Deeper Exploration of Effect Modification Findings

The reviewer's suggestion regarding deeper exploration of the subgroup findings, particularly the stronger effects observed in males <60 years, is well taken. We have substantially expanded our discussion of these findings to provide more nuanced interpretations.

In the revised Discussion section, we now:

Explore plausible sociocultural and structural explanations beyond biological mechanisms, including:

Occupational patterns and the significance of work-related social networks for middle-aged men.

Gender norms in Chinese society that may shape help-seeking behaviors and social support utilization.

Migration history patterns that may differentially affect social networks among rural residents.

Frame these explanations as hypotheses generated by our findings, explicitly noting that our data did not allow for direct testing of these mechanisms.

Include specific recommendations for future research to systematically investigate these potential pathways using both quantitative and qualitative approaches.

We believe these revisions have significantly strengthened our manuscript by more directly addressing these important methodological considerations and providing richer interpretation of our subgroup findings. The changes enhance both the scholarly rigor and the practical implications of our work.

**5. Interpretation of Mediation Results
The reported mediation effect of frailty (57.8%) is large. Please clarify whether the frailty index shares overlapping components with the physical dysfunction outcome, as this could inflate mediation estimates.
Discuss possible bi-directionality between frailty and vascular aging in more depth.**

**Response:**

We thank the reviewer for raising this important methodological concern. We have taken the following steps to address it:

Clarification on Overlapping Components:

We have explicitly clarified in the Methods section (under “Variable Definition and Measurement”) that the Frailty Index and the Physical Dysfunction outcome are constructed from non-overlapping items.

The Frailty Index was based on a cumulative deficit model incorporating 30+ health-related variables (e.g., comorbidities, cognitive status, psychological symptoms), none of which directly assess physical function.

The Physical Dysfunction outcome was defined solely based on difficulties in performing 9 basic activities of daily living (ADLs), which were not included in the Frailty Index.
This ensures that the mediation effect is not artificially inflated due to content overlap.

Additional Sensitivity Analysis:

To further validate the robustness of our mediation results, we conducted a sensitivity analysis excluding any items that might indirectly relate to physical function. The results remained consistent, confirming that the mediation effect of frailty is not driven by conceptual overlap.

Discussion on Bi-Directionality:

We have expanded the Discussion section to address the potential bi-directional relationship between frailty and vascular aging. We now cite recent longitudinal and mechanistic studies [16,67] that support a reciprocal relationship:

Vascular aging (e.g., elevated EPWV) may contribute to frailty by impairing microcirculation and promoting sarcopenia and chronic inflammation.

Conversely, frailty may accelerate vascular aging through systemic inflammation, oxidative stress, and endothelial dysfunction.
 We also acknowledge the limitations of our cross-sectional design in establishing temporal precedence and call for longitudinal studies to clarify the directionality of this relationship.

16.	Álvarez-Bustos A, Carnicero JA, Rodríguez-Sánchez B, El-Assar M, Rueda R, Pereira SL, et al. Association between pulse wave velocity and frailty, disability, and mortality in community-dwelling older adults. Jacc: Advances. 2023;2(5):100423.

67.	Hu Y, Huan J, Wang X, Lin L, Li Y, Zhang L, et al. Association of estimated carotid-femoral pulse wave velocity with frailty in middle-aged and older adults with cardiometabolic disease. Aging Clin Exp Res. 2023;35(11):2425-36.

**6.Generalizability
- As the data are from 2015, please discuss whether changes in social structures, healthcare access, or biomarker profiles in recent years might limit applicability to the current Chinese older adult population.**

**Response:**

We appreciate the reviewer’s insight regarding the temporal relevance of our findings. We have added the following discussion in the Limitations subsection of the Discussion section:

Social and Healthcare Changes:

We acknowledge that since 2015, China has undergone significant societal changes, including rapid urbanization, expansion of social insurance coverage, and the implementation of national health policies (e.g., “Healthy China 2030”). These changes may have influenced social connectivity, healthcare access, and health outcomes among older adults.

Biomarker and Disease Trends:

We note that the prevalence and management of cardiometabolic diseases (e.g., hypertension, diabetes) have evolved due to improved screening and treatment. However, the fundamental biological pathways linking social isolation, vascular aging, frailty, and physical dysfunction are likely to remain relevant.

Data Justification and Future Research:

While the CHARLS 2015 wave provides a robust, nationally representative baseline, we agree that more recent data would enhance the generalizability of our findings. We now explicitly call for replication of our analyses in more contemporary cohorts (e.g., CHARLS 2018 or 2020) to validate the stability of the observed thresholds and mediation pathways.
